# Acetylcholine Upregulates *Entamoeba histolytica* Virulence Factors, Enhancing Parasite Pathogenicity in Experimental Liver Amebiasis

**DOI:** 10.3389/fcimb.2020.586354

**Published:** 2021-01-28

**Authors:** Marina Nayeli Medina-Rosales, Martín Humberto Muñoz-Ortega, Mariana Haydee García-Hernández, Patricia Talamás-Rohana, Iliana Ernestina Medina-Ramírez, Larissa Guadalupe Salas-Morón, Sandra Luz Martínez-Hernández, Manuel Enrique Ávila-Blanco, Beatriz Medina-Rosales, Javier Ventura-Juárez

**Affiliations:** ^1^ Departamento de Morfología, Centro de Ciencias Básicas, Universidad Autónoma de Aguascalientes, Aguascalientes, Mexico; ^2^ Departamento de Química, Centro de Ciencias Básicas, Universidad Autónoma de Aguascalientes, Aguascalientes, Mexico; ^3^ Instituto Mexicano del Seguro Social, Unidad de Investigación Biomédica Zacatecas, Zacatecas, Mexico; ^4^ Departamento de Infectómica y Patogénesis Molecular, Centro de Investigación de Estudios Avanzados del Instituto Politécnico Nacional, Ciudad de México, Mexico

**Keywords:** *Entamoeba histolytica*, amebiasis, acetylcholine, virulence factors, invasiveness

## Abstract

*Entamoeba histolytica* is an invasive enteric protozoan, whose infections are associated to high morbidity and mortality rates. However, only less than 10% of infected patients develop invasive amebiasis. The ability of *E. histolytica* to adapt to the intestinal microenvironment could be determinant in triggering pathogenic behavior. Indeed, during chronic inflammation, the vagus nerve limits the immune response through the anti-inflammatory reflex, which includes acetylcholine (ACh) as one of the predominant neurotransmitters at the infection site. Consequently, the response of *E. histolytica* trophozoites to ACh could be implicated in the establishment of invasive disease. The aim of this study was to evaluate the effect of ACh on *E. histolytica* virulence. Methods include binding detection of ACh to plasma membrane, quantification of the relative expression of virulence factors by RT-PCR and western blot, evaluation of the effect of ACh in different cellular processes related to *E. histolytica* pathogenesis, and assessment of the capability of *E. histolytica* to migrate and form hepatic abscesses in hamsters. Results demonstrated that *E. histolytica* trophozoites bind ACh on their membrane and show a clear increase of the expression of virulence factors, that were upregulated upon stimulation with the neurotransmitter. ACh treatment increased the expression of L220, *Gal/GalNAc lectin* heavy subunit (170 kDa), *amebapore C*, cysteine proteinase 2 (*ehcp-a2*), and cysteine proteinase 5 (*ehcp-a5*). Moreover, erythrophagocytosis, cytotoxicity, and actin cytoskeleton remodeling were augmented after ACh treatment. Likewise, by assessing the formation of amebic liver abscess, we found that stimulated trophozoites to develop greater hamster hepatic lesions with multiple granulomas. In conclusion, ACh enhanced parasite pathogenicity by upregulating diverse virulence factors, thereby contributing to disease severity, and could be linked to the establishment of invasive amebiasis.

## Introduction

*Entamoeba histolytica*, an enteric protozoan parasite, is the causative agent of amebiasis, a worldwide-spread disease, for which 50 million new infections and over 100,000 deaths are estimated every year (Stanley, 2003; Bercu et al., 2007). In general, amebic infections are asymptomatic; however, less than 10% of the infected patients develop extraintestinal disease (Walsh, 1986). This data suggests that most of the population might carry commensal trophozoites, which under certain stimuli or environmental conditions could switch from a commensal to a pathogenic stage.

The life cycle of *E. histolytica* begins when infective cysts are ingested and travel through the gastrointestinal tract until reaching the colon; then excystation occurs and the trophozoites colonize the gut lumen. This step is thought to be critical, in which the ameba can behave as a commensal or pathogen (Ali et al., 2012). When a commensal relationship is established, it is more likely that trophozoites inhabit the gut lumen. Conversely, in the pathogenic state, the parasite degrades the colonic mucosa and encounters epithelial cells, thus enabling trophozoite adhesion and damage of the intestinal parenchyma, and finally allowing trophozoites penetration of the lamina propria (Wilson et al., 2012). Tissue damage is mainly caused by host cell death, invasion, and destructive inflammatory response. The ameba adapts rapidly to different environments; hence, even though tissue damage induces a robust immune response, inflammation favors sustained parasitic infection and exacerbates the disease (Ghosh et al., 2019). Furthermore, although the vast majority of those infected with *E. histolytica* resist to invasive disease, hosts maintaining the parasite under control show symptoms with different degrees of severity. However, since the signals that can lead to a symptomatic *E. histolytica* infection are unknown, alterations in host response to the pathogen could explain such variety in disease evolution (Nakada-Tsukui and Nozaki, 2016). In summary, acute amebiasis could result from the parasite’s ability to respond to extracellular cues and from its rapid adaptation to environmental conditions that trigger the ameba’s invasive behavior (Labruyère et al., 2019).

It is well known that the host organism can induce multiple responses to overcome infection and prevent spread. For instance, it is increasingly acknowledged that, to facilitate pathogen clearance, the immune system also communicates with the nervous systems to amplify the local immune response (de Jonge, 2013). Thereafter, the nervous system suppresses the immune response to restore homeostasis, through systemic endocrine regulation or *via* regional neuronal response through the sympathetic and parasympathetic systems (Kawli et al., 2010). The gut-brain axis is a complex and bidirectional connection between the central nervous system and the enteric nervous system. The latter is a network of intrinsic nerves within the wall of the gastrointestinal tract, spanning from the middle esophagus to the colon, and comprises primary afferent neurons, sensing mechanical and chemical stimuli; interneurons, connecting primary afferent neurons to effector cells; and motor neurons, that terminate at various points in the muscle layer and the mucosal epithelium, and innervate smooth muscle, endocrine and secretory cells (Berthoud and Neuhuber, 2000; Costa et al., 2000; Nezami and Srinivasan, 2010; Furness et al., 2014). The predominant neurotransmitter of the enteric nervous system is acetylcholine (ACh), released from parasympathetic and intrinsic nerves (Agostoni et al., 1957). ACh-releasing cholinergic fibers carried in the vagus nerve innervate the stomach, small intestine, and proximal colon (Zheng and Berthoud, 2000; Browning and Travagli, 2014). ACh has a physiologically relevant function in the intestine, regulating smooth muscle contraction, blood vessel dilation, epithelial transport, enteric hormone release, and mucus secretion (Specian and Neutra, 1980; Barry et al., 1995; Keely, 2011). Moreover, previous reports indicate that ACh also plays an important role in modulating the immune response in the gastrointestinal tract. In particular, ACh displays an anti-inflammatory effect by binding the alpha 7 (α7) subunit of the nicotinic ACh receptor (Rosas-Ballina et al., 2011), thus inhibiting monocyte and macrophage activation and reducing the production of proinflammatory cytokines (Wang et al., 2003).

Little is known about the role of ACh throughout *E. histolytica* infection. However, evidence has been provided that the vagus nerve regulates local inflammation during the development of amebic hepatic abscess. In fact, vagotomized hamsters showed a significant increase in proinflammatory cytokines induced primarily by the degradation of the NF-κB inhibitor, leading to the activation of NF-κB and STAT3 phosphorylation (Yoshikawa et al., 2006; Van Der Zanden et al., 2009; Peña et al., 2010; Sánchez-Alemán et al., 2015). Therefore, inhibition of vagal stimulation protects the host against the ameba in acute stages of the disease by magnifying the inflammatory response at the infection site and thus supporting parasite elimination (Muñoz-Ortega et al., 2011). Conversely, the presence of ACh at the infection site induces an anti-inflammatory response that could favor the evolution of amebiasis or even lead to the development of invasive disease, due to the hindering of trophozoite clearance by a retarded immune response.

In addition, previous reports showed that ACh could enhance *E. histolytica* pathogenicity. Indeed, when rats infected by intracecal inoculation with *E. histolytica* strains isolated from feces of patients were treated with physostigmine salicylate, a reversible acetylcholinesterase inhibitor that effectively increases ACh concentration, three out of five strains caused a higher mean score of cecal lesions with respect to the control group (Kulkarni and Sen, 1986). This result suggested that increased levels of ACh could modulate amebic virulence. However, the conditions and mechanisms that could contribute to such modulation are still unknown. Therefore, the aim of this study was to evaluate the possible modulatory effects of ACh on *E*. *histolytica* virulence. Although invasive amebiasis could be associated to the parasite’s virulence state, together with environmental conditions and host susceptibility, the present study demonstrates that ACh upregulates *E. histolytica* virulence factors, thereby promoting phagocytosis, cytopathic effect on liver cells, and liver abscess formation in a hamster model.

## Materials and Methods

### *E. histolytica* Culture

*E. histolytica* HM-1:IMSS trophozoites were grown under axenic conditions in TYI-S-33 medium supplemented with 10% adult bovine serum (ABS), penicillin (100 U/ml), and streptomycin (100 μg/ml) at 37°C (Diamond et al., 1978). After 72 h, during the logarithmic growth phase, tubes were chilled on ice and centrifuged for 20 min at 300 × *g*. Trophozoites were then harvested in serum free media (SFM) and counted with a hemocytometer. Finally, cell viability was evaluated using the trypan blue exclusion technique and the concentration of the cell suspension was adjusted to 1 × 10^6^ trophozoites/ml.

### Viability and Proliferation Assay

Trophozoites viability was evaluated after 1 h treatment with ACh 100, 1, 0.01, 0.0001, or 0.000001 µM. As a positive control 10 µg/ml of metronidazole was used. Viability of cells was assessed by trypan blue exclusion. Approximately 1 × 10^4^ trophozoites in the logarithmic growth phase were inoculated into 7 ml of fresh culture medium, then were observed, and counted every 24 h using a Carl Zeiss 398 Axiovert 40CFL Microscope (Carl Zeiss AG, Germany). A count was made of 100 *E. histolytica* trophozoites per condition and the average percentage of viable trophozoites was determined in three independent assays. Images were processed using Image-Pro Plus 4.5.0.19 (Media Cybernetics, Rockville, MD, USA).

### Immunofluorescence Assays

Trophozoites (2 × 10^5^) were placed on glass coverslips in the bottom of each well of a 24-well plate and incubated for 20 min at 37°C, then washed with Phosphate Buffer Solution (PBS). Afterwards, ACh 1 or 0.01 µM was added and the plate was further incubated for 1 h at 37°C. Cells were then washed with PBS, fixed with 2% paraformaldehyde (PFA) and permeabilized with 0.2% Triton X-100 in PBS. For ACh detection, a 1 h incubation with anti-acetylcholine-FITC polyclonal antibody (1:800; LS−C305726, LifeSpan BioSciences, Seattle, Washington, USA) was performed. For double immunodetection, trophozoites were treated with Alexa Fluor 647-conjugated α-bungarotoxin (1:100; B35450, Invitrogen, Carlsbad, California, USA) or primary polyclonal antibody against L220 (1:500) incubated for 1 h, then washed with PBS and further incubated with secondary antibody Alexa Fluor 594 conjugated goat anti mouse-IgG (H+L) (1:1000; A-11005, Invitrogen, Eugene, Oregon, USA). Cells were then washed and incubated with 1 μg/ml Hoechst 33342 (Sigma‐Aldrich, Poole, Dorset, UK) for 10 min for nuclear staining. Subsequently, trophozoites were washed with PBS and fixed with 2% PFA. Finally, coverslips were mounted with Vectashield (Vector Laboratories, Burlingame, California, USA) and observed with a Carl Zeiss LSM 700 Laser Scanning Microscope (Carl Zeiss AG) with a X63 oil immersion objective. Images were acquired using the Zen Black 2012 (black edition) software (ZEISS). Image analysis to determine colocalization between ACh and L220 was performed through the ImageJ software (Wayne Rasband, Nat. Inst. of Health, USA). Analysis was performed on a similar sized region of interest selected for each channel. The comparative degree of colocalization was calculated as mean Pearson’s and Mander’s R coefficients on the red and green channel, considering R values above 0.6 threshold values as significant.

### RT-qPCR

Total RNA from 1 × 10^6^ trophozoites incubated or not with ACh 1, 0.01, 0.0001, or 0.000001 µM for 1 h was isolated with the Direct-zol RNA Miniprep kit (Zymo Research, Irvine, California, USA), following the manufacturer’s protocol. Reverse transcription was performed with 500 ng of total RNA from *E. histolytica* using the Revert Aid First Strand cDNA Synthesis Kit (Thermo Fisher Scientific), and gene expression was measured using 50 ng of cDNA by quantitative real-time PCR with the Maxima SYBR Green qPCR Master Mix (2×) (Thermo Scientific, California, USA) in a Step One machine (Applied Biosystems, Thermo Fisher Scientific, California, USA) using the following program: 50°C for 2 min, 95°C for 3 min, and 40 cycles of 95°C for 30 sec and 56°C for 30 sec. Oligonucleotides were designed to target genes encoding *E. histolytica* cysteine proteinase 2 (*ehcp-a2*), cysteine proteinase 5 (*ehcp-a5*), *amebapore C*, and *Gal/GalNAc lectin* heavy subunit (**Table 1**). Expression levels were normalized to that of the housekeeping gene *α-tubulin* and differences were determined by employing the 2^-ΔΔCt^ method (Livak and Schmittgen, 2001), using the StepOne machine.

**Table 1:** *Entamoeba histolytica* gene-targeted primers used in this study.

Target	Sequence (5´→3´)
Cysteine Protease 2 (*ehcp-a2*)	Fwd: TGGACCATTTGCTGCTATGA Rev: TAACATGATCCGCATTGTGC
Cysteine Protease 5(*ehcp-a5*)	Fwd: AATTCATGGGGAACTATTTGG Rev: CATCAGCAACCCCAACTGG
Amebapore C	Fwd: TCCTCTGCAACCTTTGCACT Rev: GCACAAATAGCATTGGCATCA
Gal/GalNAc lectin heavy subunit (170 kDa)	Fwd: TGACCTTGGTATTATGTCTCG Rev: GTCTCCATGGTTGCATAGC
α-tubulin	Fwd: TGCACCAATTGTTACACCAGA Rev: CATGGACACCATCCAACAAA

### Western Blot

After incubation, trophozoites were washed with PBS. For protein extraction cells were lysed in RIPA buffer (Sigma-Aldrich, St Louis, Missouri, USA) with protease inhibitors (50 mM Tris-HCl, pH 6.8, 5 mM N-ethylmaleimide, 3 mM iodoacetamide, 1 mM phenylmethanesulfonyl fluoride, and 3 mM tosyl-l-lysine chloromethyl ketone) at 4°C for 30 min. Protein quantification was performed with the Bradford method (Bradford, 1976). For Western blotting, 50 µg of each protein extract was separated in a 10% SDS-PAGE gel, and proteins were transferred (12 mA, low voltage overnight at 4°C) to polyvinylidene difluoride (PVDF) membranes (Bio-Rad, Hercules, CA, USA). The membranes were blocked with Tris-buffered saline (TBS) and 5% skimmed milk for 1 h at room temperature. For immunodetection, the membranes were incubated for 24 h at 4°C with the primary antibody, rabbit polyclonal antibody anti-β-actin (1:1,000; ab8227, Abcam, Cambridge, UK), polyclonal monospecific mouse anti-L220 or monoclonal mouse anti-α-tubulin (1:2,000; T6074, Sigma, USA). Blots were incubated for 2 h at room temperature with goat anti-mouse IgG-HRP conjugated (1:2,000; AP127P, Chemicon, USA) or goat anti-Rabbit IgG-HRP conjugated (1:2,000; A0545, Sigma, USA). After the incubation, the membranes were washed with TBST (Tris-buffered saline– 0.05% Tween 20) and blots were revealed with Clarity Western ECL substrate (Bio-Rad, Hercules CA, USA) for chemiluminescence imaging.

### Cysteine Protease Activity

Evaluation of the intracellular cysteine protease activity in total amebic lysates of treated trophozoites with E-64, ACh 1, 0.01, 0.0001, or 0.000001 µM as previously described (Bosch et al., 2012; Dolabella et al., 2012). 100 µg of amebic extract and 2 mg of azo dye-impregnated collagen (Sigma-Aldrich, St Louis, Missouri, USA) resuspended in 500 µL of protease activation buffer (100 mM Tris pH 7.0 and 10 mM CaCl2), were incubated at 37°C for 18 h. After this reaction was stopped by adding 500 µL of 10% TCA. Then samples were centrifuged, collagen fibers were discarded, and supernatants collected for spectrophotometric determination at 540 nm.

### Polymerized Actin Detection Assay

Trophozoites were stained with primary polyclonal antibody against β-actin (1:1000; ab8227, Abcam, Cambridge, UK) incubated for 1 h, then washed with PBS and further incubated with secondary antibody Alexa Fluor 488 conjugated goat anti rabbit-IgG (H+L) (1:1000; A-11008, Invitrogen, Eugene, Oregon, USA). Then 6 μM (1 UI/ml) rhodamine-phalloidin (Molecular probes, Eugene, Oregon, USA) were incubated for 30 min and Hoechst 33342 for 10 min for nuclear staining. Before this step, trophozoites were treated with ACh 1, 0.01, 0.0001, 0.000001, or 1 µM cytochalasin D (CD, Thermo Fisher Scientific, Waltham, Massachusetts, USA), then fixed with 2% PFA and permeabilized with 0.2% Triton X-100 in PBS. Finally, slides were mounted in Mowiol (Sigma-Aldrich, St Louis, Missouri, USA) and examined by confocal microscopy using Carl Zeiss LSM 700 Laser Scanning Microscope (Carl Zeiss AG) with a 63× oil immersion objective. Images were acquired using the Zen Black 2012 (black edition) software (ZEISS). Quantification of fluorescence intensity of fibrillar actin (F-actin) and globular actin (G-actin) structures was performed with the ImageJ software (Wayne Rasband, Nat. Inst. of Health, USA). Images were converted to 8-bit greyscale and mean fluorescence intensity was measured and expressed as the mean grey value, that is defined as the average grey value for all pixels within the indicated area.

### Cytotoxicity Assay

HepG2 cells were grown in a 24-well plate until reaching a 90% confluent monolayer in minimum essential medium (DMEM, Thermo Fisher Scientific), and trophozoites (2 × 10^4^/well) non treated or pre-treated with E-64, ACh 1, 0.01, 0.0001, or 0.000001 µM for 1 h, or their supernatants were distributed into each well. Plates were then incubated for 2 h at 37°C. Afterward, trophozoites were removed from the monolayer by placing the 24-well plate on ice for 20 min and washing the wells with cold PBS. After this step, the remaining monolayer cells were fixed with 2% PFA for 20 min and stained with 0.1 M methylene blue in borate buffer (pH 8) for 15 min. To remove excess dye, cells were washed twice with 0.1 M borate buffer. For dye extraction, each well was treated with 1 ml of 1 N hydrochloric acid (HCl) for 30 min at 37°C. To determine absorbance at 655 nm, samples were analyzed in a microplate spectrophotometer (Microplate Reader, iMark, BioRad, Hercules, CA, USA). The percentage of monolayer damage was calculated as:

$$[{\text{OD}}_{655}\left(\text{control wells}\right)-{\text{OD}}_{655}\left(\text{experimental wells}\right)/{\text{OD}}_{655}\left(\text{control wells}\right)]\times 100$$

### Erythrophagocytosis

*E. histolytica* trophozoites (2 × 10^5^) were transferred onto glass coverslips placed in the bottom of each well of a 24-well plate, then incubated for 15 min at 37°C in order to allow adhesion to the coverslip surface, and subsequently treated with ACh 1 or 0.01 µM, for 1 h. Following incubation, trophozoites were washed twice with PBS before the assay. Fresh human erythrocytes in PBS solution were stained with phycoerythrin (PKH26, Sigma-Aldrich, St Louis, Missouri, USA), counted, and used at a 1:20 trophozoites:erythrocytes ratio. To establish the interaction, erythrocytes were added to trophozoites, and incubation was carried out for 20 min at 37°C on SFM, followed by two rounds of washing with PBS. Lysis buffer (0.15 M ammonium chloride (NH_4_Cl), 10 mM potassium bicarbonate (KHCO_3_), and 0.1 mM EDTA) was added for 1 min at room temperature to remove non-phagocytosed red blood cells. Afterwards, 0.5 ml of fetal bovine serum (FBS, GIBCO, Grand Island, New York, USA) was added for 1 min at room temperature and cells were washed once with PBS. Trophozoites were then fixed with 2% PFA for 20 min at room temperature and washed with PBS, and nuclei were stained with 1 μg/ml Hoechst 33342. Finally, samples were mounted in Mowiol and examined by epifluorescence microscopy (Axioskop 40). Images were processed using Image-Pro Plus 4.5.0.19.

### Indirect Determination of Erythrophagocytosis

For the indirect quantification of erythrocytes ingested by the ameba; hemoglobin was determined by a colorimetric method. Trophozoites (2 × 10^5^) were treated with ACh 1, 0.01, 0.0001, or 0.000001 µM for 1 h. Then trophozoites were washed in PBS solution and incubated with erythrocytes (2 × 10^7^) for 20 min at 37°C on SFM. After incubation, 1 ml of cold distilled water was added to lyse non-engulfed erythrocytes and centrifuged for 5 min at 700 x g, pellet was resuspended in 1 ml of acetic acid (CH₃COOH) 2% solution to lyse remaining free erythrocytes followed by centrifugation 5 min at 700 x g. Then 1 ml of formic acid (CH₂O₂) was added to burst trophozoites. Hemoglobin was measured by spectrophotometric analysis at 405 nm.

### Transwell Migration Assay

To evaluate the effect of ACh on trophozoite migration, cells were washed and resuspended in plain TYI-S-33 medium without addition of vitamins and serum. 5 × 10^4^ trophozoites were added to the top of a transwell insert containing 8-μm pores (Costar, Cambridge, Massachusetts, USA). Chemoattractant gradients were generated by placing 600 µL of culture SFM containing ACh 1, 0.01, 0.0001, 0.000001 µM, or interferon gamma (IFN-γ), interleukin-8 (IL-8), and 20% ABS in the lower chamber of the migration units. To inhibit chemotaxis, trophozoites were treated with CD. The 24-well plate was kept for 2 h at 37°C and 0.05% CO_2_. Cells that migrated to the lower surface of the membrane were fixed with 4% PFA and stained with 1 μg/ml Hoechst 33342 for 20 min. Trophozoite migration was then determined by counting the number of trophozoites that were attached to the bottom of the well with a Carl Zeiss Axiovert 40CFL microscope. Images were processed with the AxioVision 40V 4.6.3.0 software (Göttingen, Germany). Chemotaxis gradient assay was performed to evaluate individual migrating trophozoites, 50 µl of ACh 0.01, 0.0001, or 0.000001 µM were injected into a 0.75% agarose gel, trophozoites were placed around the impregnated ACh agarose gels, and then incubated for 15 min at 37°C. Chemotaxis of trophozoites toward ACh was visualized using phase-contrast video microscopy. Video registers were made with a Carl Zeiss Axiovert 40CFL inverted microscope. Time-lapse videos were generated from 114 spaced frames, with 2 s between each frame acquired for 4-min real-time registers. Each video was processed with AxioVision 40V 4.6.3.0 software. To register random motility of trophozoites in the absence of ACh, SFM was used as a control, additionally, CD was used as a negative control. The data analysis was performed using ImageJ (Wayne Rasband, Nat. Inst. of Health, USA).

### Experimental Amebic Liver Abscess

To induce amebic liver abscess (ALA), 20 male golden hamsters (*Mesocricetus auratus*) were grouped in normal, sham, *E. histolytica* and *E. histolytica* + ACh (5 per each group). Livers were inoculated with 7.5 × 10^5^ trophozoites that had been incubated with or without 0.0001 µM ACh for 1 h. After incubation, trophozoites were washed with PBS, suspended in 100 μl of culture medium and injected into the right liver lobe of hamsters. Sham-treated control hamsters were injected with culture medium only. The animals were sacrificed after 4 d by anaesthetization with sodium pentobarbital (50 mg/kg, i.p.), and livers were excised for macroscopic evaluation. Samples of liver tissues were dissected and fixed in 4% PFA in PBS, embedded in paraffin, and processed by conventional histological techniques to obtain tissue slices. Afterwards, samples were deparaffinized and rehydrated with PBS. Liver tissue slides were stained with hematoxylin & eosin for identification of infiltrated inflammatory cells and quantification of tissue damage area.

### Statistical Analysis

For statistical analysis, the GraphPad Prism 5.0 software was used (San Diego, California, USA). The Shapiro-Wilk test was used to verify the normality of data. Differences between two groups were assessed by unpaired Student t-test. Analysis of variance was performed either with Kruskal-Wallis test and Dunn post-hoc test or with one-way ANOVA and Tukey post-hoc test, depending on data distribution. A value of p < 0.05 was considered as threshold for significant differences (*).

## Results

### Effect of Acetylcholine on Viability and Proliferation of *E. histolytica*

**Figure 1:**
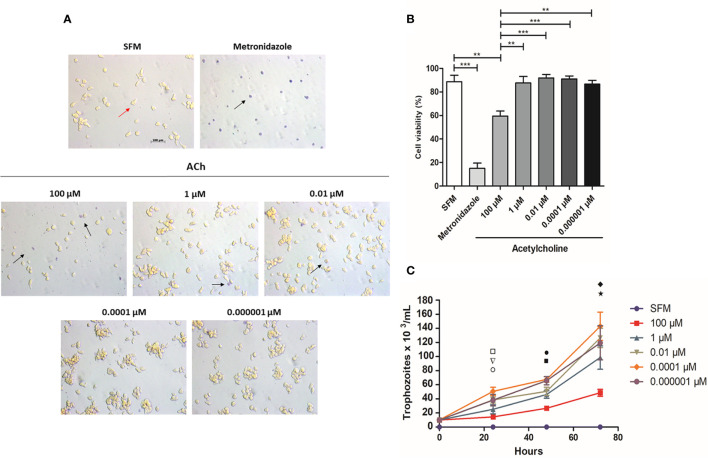
Viability of *E. histolytica* trophozoites using trypan blue dye exclusion test. **(A)** Representative images after 1 h ACh treatment with different concentrations (red arrow, viable trophozoites; black arrow, dead trophozoites). **(B)** Cells viability after 1 h ACh treatment of trophozoites. **(C)** Proliferation of *E. histolytica* trophozoites upon ACh treatment. Trophozoites were counted every 24 h. Statistical significance comparing means pairwise: □ SFM vs 0.0001 µM (**), ▽ 1 µM vs 0.0001 µM (**), ○ 100 µM vs 0.0001 µM (*), ▪ 100 µM vs 0.0001 µM (***), • 100 µM vs 0.000001 µM (***), ♦ SFM vs 100 µM (*) and ⋆ 100 µM vs 0.0001 µM (**). Representative images of the analysis by light microscopy at X10 are shown. Data correspond to the mean ± SEM of three independent experiments (n = 3). The statistical analysis was performed with the Kruskal Wallis and Tukey posttest method, where the values of *p < 0.05, **p < 0.01 and ***p < 0.001 were considered significant.

Trophozoite viability after ACh treatment was evaluated. Interestingly, there was no difference between treated and untreated trophozoites except for those stimulated with 100 µM ACh, which displayed reduced viability (59.50 ± 8.544%), also presenting a rounder shape and smaller size when compared to non-treated cells (**Figure 1A**). On the other hand, 1, 0.01, 0.0001, and 0.000001 µM ACh had no effect on trophozoite viability when compared to SFM control (**Figure 1B**). To evaluate the effect of ACh on trophozoite proliferation we performed a proliferation assay, ACh treated cells were cultured in fresh medium and counted at 24, 48, and 72 h. 0.0001 µM ACh induced a significant increase of trophozoites number at 24 h in relation to the control. Contrary to this 100-µM ACh reduced proliferation significantly at 72 h, important to mention that 100 µM ACh stimulus, also showed a clear tendency to diminished proliferation at 24 h and 48 h when compared to SFM control. Otherwise, 1, 0.01, 0.0001, and in 0.000001 µM ACh showed slight tendency to increase trophozoite proliferation (**Figure 1C**).

### Acetylcholine Detection on *E. histolytica*

**Figure 2:**
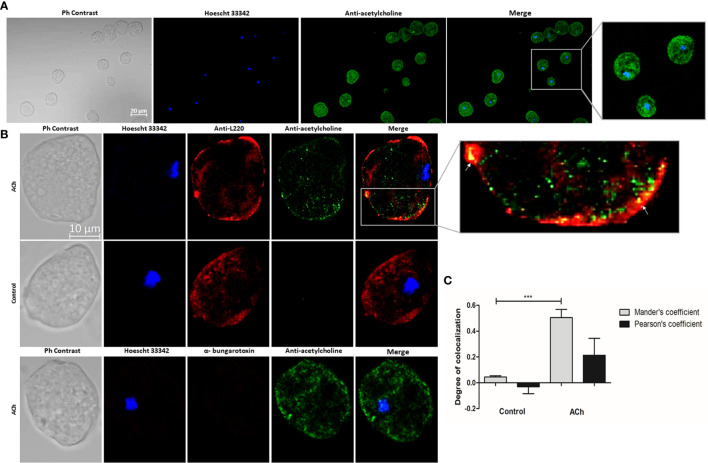
Acetylcholine binding on E. histolytica membrane. **(A)** Trophozoites treated with 1 µM ACh for 1 h were fixed and permeabilized. For immunodetection, anti-acetylcholine-FITC (green) antibody (1:800 dilution) and Hoechst 33342 (blue) for nuclear staining were used. Images were obtained by confocal microscopy at X20. **(B)** Cellular location of ACh and L220 (as a surface marker) on E. histolytica trophozoites membrane by using anti-acetylcholine-FITC (green) antibody (1:800 dilution), and an anti-L220 (red) antibody (1:500 dilution). Additionally, α-bungarotoxin-Alexa Fluor 647 (1:100) was also used. Nuclei were counterstained with Hoechst 33342 (blue). Representative images of the confocal microscopy analysis at X63. **(C)** Colocalization between ACh and L220 quantified and compared using Pearson’s and Mander’s correlation coefficients (considering as significant R coefficients values above 0.6 threshold). Data correspond to the mean ± SEM of five independent experiments (n = 5). The statistical analysis was performed with the one-way ANOVA and Tukey posttest method, where the values of ***p < 0.001 were considered significant.

ACh binding to the parasite’s membrane was detected (**Figure 2A**). To confirm ACh location on the surface of trophozoite membranes, we used an antibody against the *E. histolytica* 220-kDa lectin (L220), a membrane protein with lectin properties involved in adherence to cells or the extracellular matrix (Rosales-Encina et al., 1987) (**Figure 2B**). Image analysis showed false overlaps between red and green pixels (Dunn et al., 2011). These results demonstrated that the ameba binds ACh and that this interaction takes place on the trophozoite membrane. But Pearson’s and Mander’s correlation coefficient demonstrate a low pixel colocalization, therefore, this association is not considered as a true interaction between L220 and ACh (**Figure 2C**). With the purpose to identify the kind of receptors involved in the ACh binding, we applied α-bungarotoxin-Alexa Flour 647, a neurotoxin that specifically inhibits nicotinic receptors; we were not able to detect any signal of Alexa Flour 647 (**Figure 2B**). In this sense, we identified an association between ACh and *E. histolytica* trophozoites membrane, but we do not know the type of interaction or molecules involved in this ACh binding. Based on these results we focused on the effect of the neurotransmitter on *E. histolytica* inducible pathogenetic factors.

### Acetylcholine Upregulates the Expression of *E. histolytica* Virulence Factors

**Figure 3:**
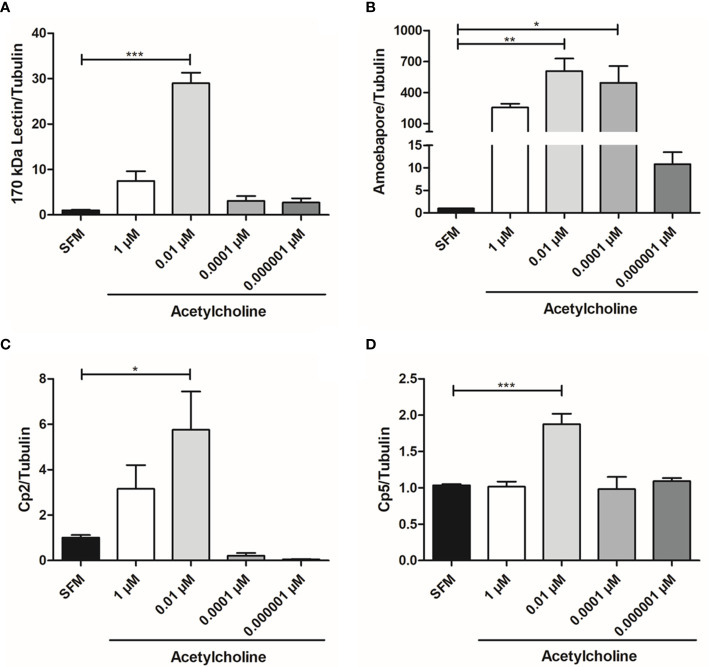
Acetylcholine upregulates *E. histolytica* virulence factors expression. Relative expression of the **(A)** *Gal/GalNAc lectin* heavy subunit, **(B)** *Amebapore*, **(C)** *ehcp-a*2 and **(D)** *ehcp-a*5 by RT-qPCR amplification from treated trophozoites with different concentrations of ACh for 1 h. Relative expression levels normalized with constitutive gene *α-tubulin*. Data correspond to the mean ± SEM of three independent experiments (n = 3). The statistical analysis was performed with the one-way ANOVA and Tukey posttest method, where the values of *p < 0.05, **p < 0.01 and ***p < 0.001 were considered significant.

The relative expression of gene *Gal/GalNAc lectin* heavy subunit (170 kDa), *ehcp-a2*, *ehcp-a5*, and *amebapore C* in trophozoites treated with 1, 0.01, 0.0001, or 0.000001 µM ACh for 1 h was evaluated by RT-qPCR (**Figure 3**). Expression levels were normalized with those of the constitutive gene α-*tubulin.* ACh upregulated the expression of *E. histolytica* virulence factors. In fact, the mRNA levels of *ehcp-a2*, *ehcp-a5*, *amebapore C*, and *Gal/GalNAc lectin* heavy subunit significantly increased after 1 h exposure to 0.01 µM ACh, as compared to non-treated trophozoites. The increase was highest for the *Gal/GalNac lectin* subunit and *amebapore C*, which presented a 28.96- and 606-fold increase, respectively (**Figures 3A, B**), with respect to the control. Meanwhile, *ehcp-a2* and *ehcp-a5* augmented 5.7- and 6.9-fold (**Figures 3C, D**). Furthermore, we also observed that 0.0001 µM ACh treatment, positively influenced *amebapore C* expression, with a 494-fold increase with respect to the untreated control (**Figure 3B**). To validate these results, we performed western blot analysis for L220 and β-actin, and a spectrophotometric method to evaluate cysteine proteases (CP) activity.

### Protein Expression and Cysteine Protease Activity

**Figure 4:**
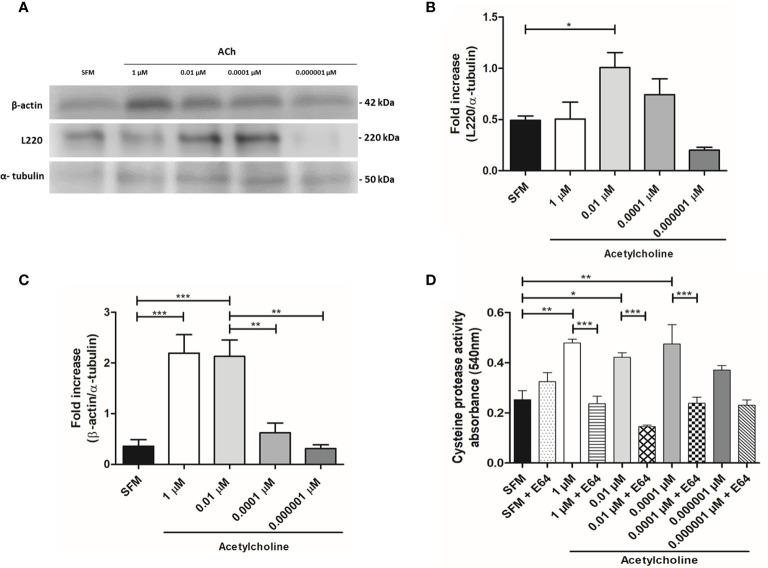
Expression of β-actin and L220 analyzed by western blot, and cysteine protease activity evaluation in ACh-treated trophozoites. **(A)** Immunodetection from total lysates of trophozoites treated with different concentration of ACh for 1 h, using anti-β-actin, anti-L220 and anti-α-tubulin antibodies in Western blot assays. **(B)** Densitometric analysis L220 and **(C)** β-actin using ImageJ software. Protein levels were normalized to α-tubulin. **(D)** Intracellular cysteine protease activity assessed by a spectrophotometric azo-collagen assay. Total extracts of ACh-stimulated trophozoites in the presence or absence of E-64 (a cysteine protease inhibitor). Data correspond to the mean ± SEM of three independent experiments (n = 3). The statistical analysis was performed with the one-way ANOVA and Tukey or Dunnett’s posttest method, where the values of *p < 0.05, **p < 0.01 and ***p < 0.001 were considered significant.

*E. histolytica* hallmarks of virulence include adhesion, phagocytosis, and secretion of soluble factors, being L220, cytoskeleton rearrangement and CP activity representative indicators of pathogenicity, respectively. Taking this in consideration, we evaluated the protein expression of L220 and β-actin in ACh stimulated trophozoites (**Figure 4A**). Our results demonstrated that 0.01 µM ACh can induce a significant augment of L220 expression (**Figure 4B**). This could be indicating that ACh favors the adhesion of trophozoites for a greater cytopathic effect. Many biological process of *E. histolytica* are actin cytoskeleton dependent including motility, phagocytosis, and secretion (Manich et al., 2018). Consequently, we analyzed the changes in trophozoite actin content under the presence of ACh. Western blot analysis showed that expression of β-actin increased at 1 and 0.01 µM ACh concentrations after 1 h treatment (**Figure 4C**), suggesting that this neurotransmitter could be participating in a cytoskeleton rearrangement mechanism that has not been elucidated yet under ACh stimulation. Other important mechanism in cytotoxic effect of *E. histolytica* is the secretion of soluble factors as CP and amebapores, through a spectrophotometric assay we detected that the activity of CP of ACh treated trophozoites is higher in those trophozoites stimulated with ACh 1, 0.01, and 0.0001 µM when comparing to the control (**Figure 4D**). These data have proven that ACh can modulate different actin dependent cellular process in *E. histolytica* trophozoites.

### Actin-Rich Structures

**Figure 5:**
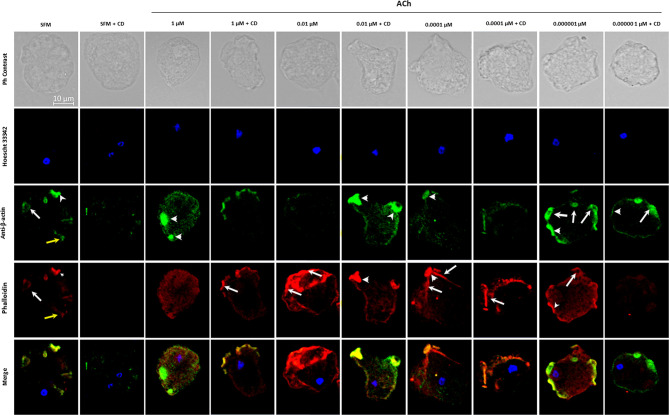
Detection and location of G-actin and F-actin in trophozoites treated with acetylcholine. G-actin was determined by using anti-β-actin, followed by antibody Alexa Fluor 488 conjugated goat anti rabbit-IgG (1:1000) (green); F-actin was determined with rhodamine-phalloidin (red); and nuclear DNA staining was done by Hoechst 33342 (blue). Cytochalasin D (CD) was used as a negative control. Representative images acquired by confocal microscopy at X63 show F-actin rich structures as macropinosomes (white arrows), adhesion plates (white arrow heads) and F-actin cytoplasmic dots (yellow arrows).

**Figure 6:**
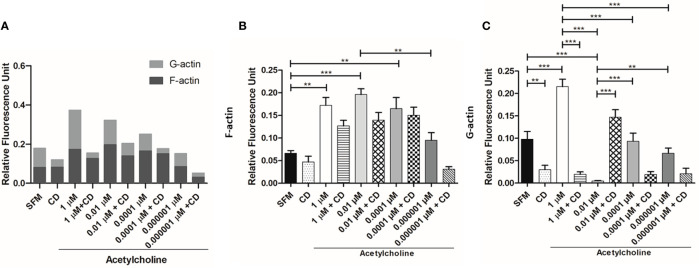
Quantitative analysis of fluorescence intensity of F- and G-actin. Analysis of fluorescence intensity was done at the original magnification by measuring the mean gray value with ImageJ software. **(A)** Combined determination of F- and G-actin in ACh-treated trophozoites. Determination of FMI for **(B)** F-actin and **(C)** G-actin. Data correspond to the mean ± SEM of three independent experiments (n = 3). The statistical analysis was performed with the Kruskal Wallis and Dunn posttest method, where the values of **p< 0.01 and ***p < 0.001 were considered significant.

To evaluate the changes and distribution of amebic cytoskeleton in *E. histolytica* trophozoites after 1, 0.01, 0.0001, 0.000001 μM ACh treatment, the presence of G-actin and F-actin in ACh stimulated trophozoites was analyzed by confocal microscopy. Cytoskeleton rearrangement primarily occurs through actin polymerization, forming different actin rich structures. In this study, we identified monomeric or globular actin (G-actin) using an anti-β-actin antibody and polymerized or fibrillar actin (F-actin) using rhodamine-phalloidin. SFM trophozoites showed a basal state, where structures like macropinosomes, adhesion plates and some F-actin dots are observed. Cells pre-incubated with 1 μM ACh, presented a higher quantity of G-actin in structures resembling adhesion plates, and a lower presence of F-actin distributed along the cytoplasm, when compared to control trophozoites. In the case of 0.01-μm ACh stimulus, trophozoites displayed predominant F-actin signals concentrated in peripheral structures like adhesion plates, and a significant decrease in G-actin with respect to the control. In trophozoites treated with 0.0001 μM ACh, we found a low G-actin occupation, and an important presence of F-actin in macropinosomal structures. When trophozoites were incubated with 0.000001 μM ACh, they showed an increase of both, G-actin, and F-actin. F-actin shaped like adhesion plates, F-actin dots and macropinosomes increased in all ACh concentrations and their presence significantly declined when trophozoites were pre-incubated with CD (**Figure 5**). G-actin and F-actin were quantified by determining its median fluorescence intensity (MFI). Comparison of the presence of G-actin and F-actin is represented in **Figure 6A**. Statistical analysis demonstrates that 1, 0.01, 0.0001 μM ACh significantly increased actin polymerization with respect to the control (**Figure 6B**). It is remarkable that 1 μM ACh significantly increased G-actin when compared to the control. Contrary to this, 0.01 μM ACh significantly reduced G-actin, in contrast to untreated trophozoites (**Figure 6C**).

### Acetylcholine Increases *E. histolytica* Cytopathic and Cytotoxic Activity Toward HepG2 Cells

**Figure 7:**
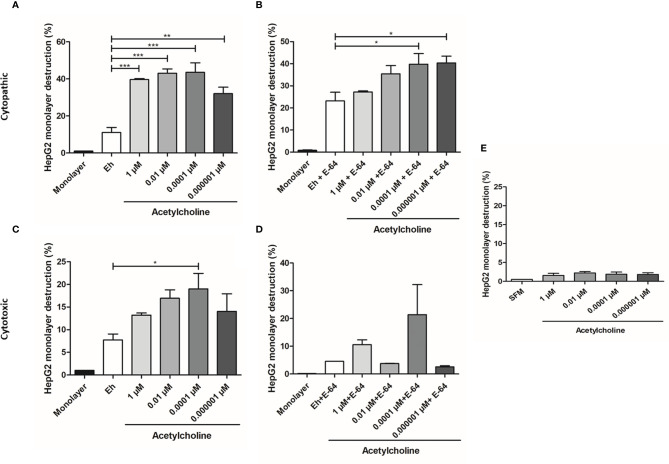
Acetylcholine-mediated cytopathic and cytotoxic effects of *E. histolytica* trophozoites. **(A)** HepG2 cells in interaction with trophozoites pre-treated with different concentrations of ACh for 1 h. **(B)** HepG2 cells in interaction with trophozoites pre-treated with E-64 and different ACh concentrations for 1 h. **(C)** HepG2 cells incubated with supernatants of *E. histolytica* trophozoites previously stimulated with ACh for 1 h. **(D)** HepG2 cells incubated with supernatants of *E. histolytica* trophozoites pre-treated with ACh and E-64 for 1 h. **(E)** HepG2 monolayer treated with ACh for 2 h as a control. Data correspond to the mean ± SEM of three independent experiments (n = 3). The statistical analysis was performed with the Kruskal Wallis and Dunn posttest method, where the values of *p < 0.05, **p < 0.01 and ***p < 0.001 were considered significant.

To determine if ACh pre-treatment increased the cytopathic activity of *E. histolytica*, monolayer destruction assay was carried out using liver HepG2 cell monolayers. *E. histolytica* trophozoites, after 1-h incubation with 1, 0.01, 0.0001, 0.000001 µM ACh, displayed a stronger cytopathic effect as compared to non-treated trophozoites (**Figure 7A**). When trophozoites where treated with both ACh and E-64 (cysteine protease inhibitor), a concentration inverse dependent monolayer destruction was observed, *E. histolytica* trophozoites stimulated with ACh 0.0001, 0.000001 µM and E-64, the percentage of monolayer destruction was significantly increased when comparing to controls (**Figure 7B**). A clear trend following a very similar pattern of monolayer damage was observed when *E. histolytica* trophozoites treated with ACh supernatants were added to HepG2 cell monolayers, demonstrated that 0.0001 µM ACh stimulation promoted contact-independent cell killing (**Figure 7C**). Something similar can be observed in monolayer destruction by supernatants of ACh and E-64 pretreated trophozoites, where cytotoxic effect independent of CP tends to increase when trophozoites were stimulated ACh 0.0001 µM (**Figure 7D**). Monolayer treated with ACh different concentrations for 2 h did not presented changes destruction percentages in relation to control (**Figure 7E**). In the present work we have demonstrated a clear cytopathic (contact-dependent) and cytotoxic (contact independent) increment in *E. histolytica* trophozoites when exposed to ACh. The mechanism by which *E. histolytica* destroys target cells can includes several processes like adhesion, cell killing and phagocytosis. Is therefore important to elucidate if ACh promotes the phagocytic capacity of the ameba.

### Erythrophagocytosis

**Figure 8:**
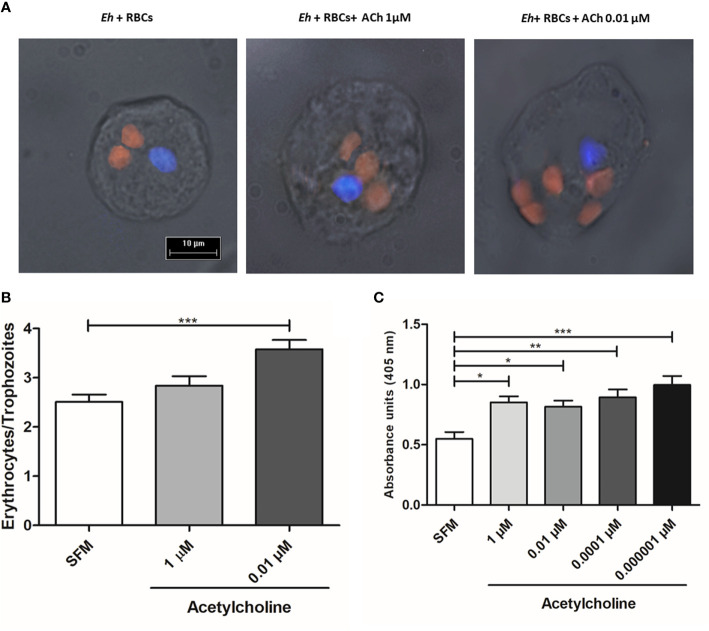
Acetylcholine increases phagocytic capacity of *E. histolytica*. **(A)** Ingested erythrocytes by trophozoites treated with ACh, evidenced by fluorescence microscopy (X40 representative images). **(B)** Mean number of phagocytosed erythrocytes by trophozoites in SFM or treated for 30 min with and 1 µM and 0.01 µM ACh. **(C)** Hemoglobin quantification by spectrophotometric analysis at 405 nm, as indirect determination of erythrophagocytosis of trophozoites treated with different concentrations of ACh for 1 h. Data correspond to the mean ± SEM of three independent experiments (n = 3). The statistical analysis was performed with the Kruskal Wallis and Dunn posttest method, where the values of *p < 0.05, **p < 0.01 and ***p < 0.001 were considered significant.

*E. histolytica* phagocytic activity has been accepted as a virulence mechanism and it is considered a central feature of intestinal invasive amebiasis (Talamás-Lara et al., 2014). 0.01 µM ACh treatment for 20 min induced a notable increase of *E. histolytica* phagocytic activity (**Figure 8A**). In fact, the number of erythrocytes ingested per trophozoite augmented significantly after ACh stimulation, with a 1.4-fold increase, in comparison to untreated trophozoites (**Figures 8A, B**). An intracellular hemoglobin spectrophotometric quantification method demonstrates that stimulated trophozoites with 1, 0.01, 0.0001, 0.000001 µM ACh presented significantly augmented hemoglobin levels when compared to non-treated controls (**Figure 8C**). These results showed that ACh stimulation can promote an increase in phagocytosis that together with migration, are essential processes for parasite proliferation and immune response evasion during the invasive process.

### Acetylcholine Promotes Chemotaxis and Migration of *E. histolytica*

**Figure 9:**
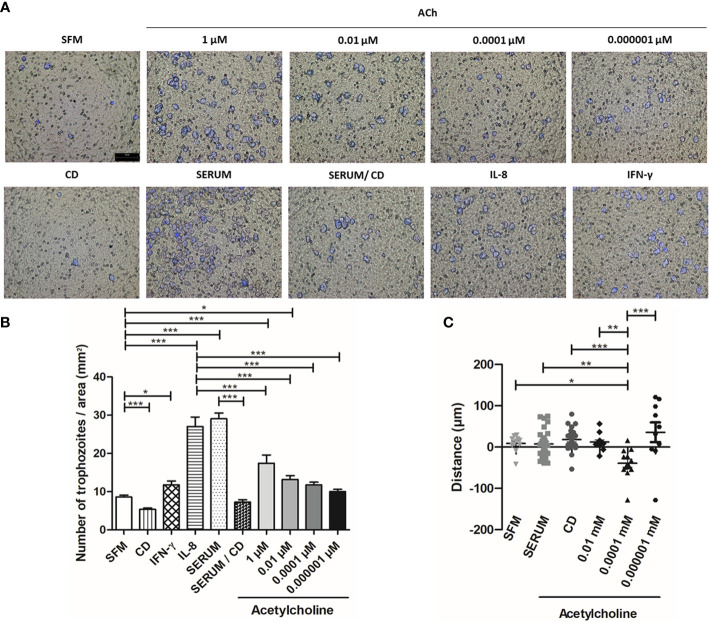
Acetylcholine induces *E. histolytica* migration and chemoattractant activity. **(A)** Representative images of the analysis by fluorescence microscopy at X10 are shown. **(B)** Number of trophozoites per area (mm^2^), that migrated through the transwell chamber in the presence of ACh, serum, IL-8 and IFN-γ. **(C)** Distance and direction of amebic movements towards ACh (**Supplementary Video**). Data correspond to the mean ± SEM of three independent experiments (n = 3). The statistical analysis was performed with the One-way ANOVA and Tukey or Kruskal Wallis and Dunn posttest method, respectively, where the values of *p < 0.05, **p < 0.01 and ***p < 0.001 were considered significant.

We then used a transwell migration assay to evaluate the effect of ACh on the migration and invasion ability of *E. histolytica* trophozoites. The migration of trophozoites in SFM was significantly enhanced upon 1 and 0.01 µM ACh treatment, as compared to untreated control trophozoites (**Figures 9A, B**). As positive control, we used interleukin-8 (IL-8), interferon gamma (IFN-γ), or fetal bovine serum (FBS). To inhibit actin cytoskeleton polymerization, CD was used as a negative control (**Figures 9A, B**). Additionally, chemotaxis of *E. histolytica* was evaluated by time-lapse video. For each recording in real time, representative *E. histolytica* trophozoites were selected for each condition, and their trajectories were followed throughout 15 min. This analysis showed that ACh 0.0001 µM is a chemoattractant to trophozoites, when comparing chemotaxis towards serum or SFM (**Figure 9C**). Thus, even though the mechanisms involved in the regulation of trophozoite motility are still under study, these results indicate that ACh 0.0001 has chemotactic effect, being 1 and 0.01 µM ACh concentrations positive regulators of *E. histolytica* migration. Therefore, it is possible that *in vivo*, this neurotransmitter could promote amebic migration, thus facilitating host invasion.

### Acetylcholine Effect on the Development of *E. histolytica* Amebic Liver Abscess

**Figure 10:**
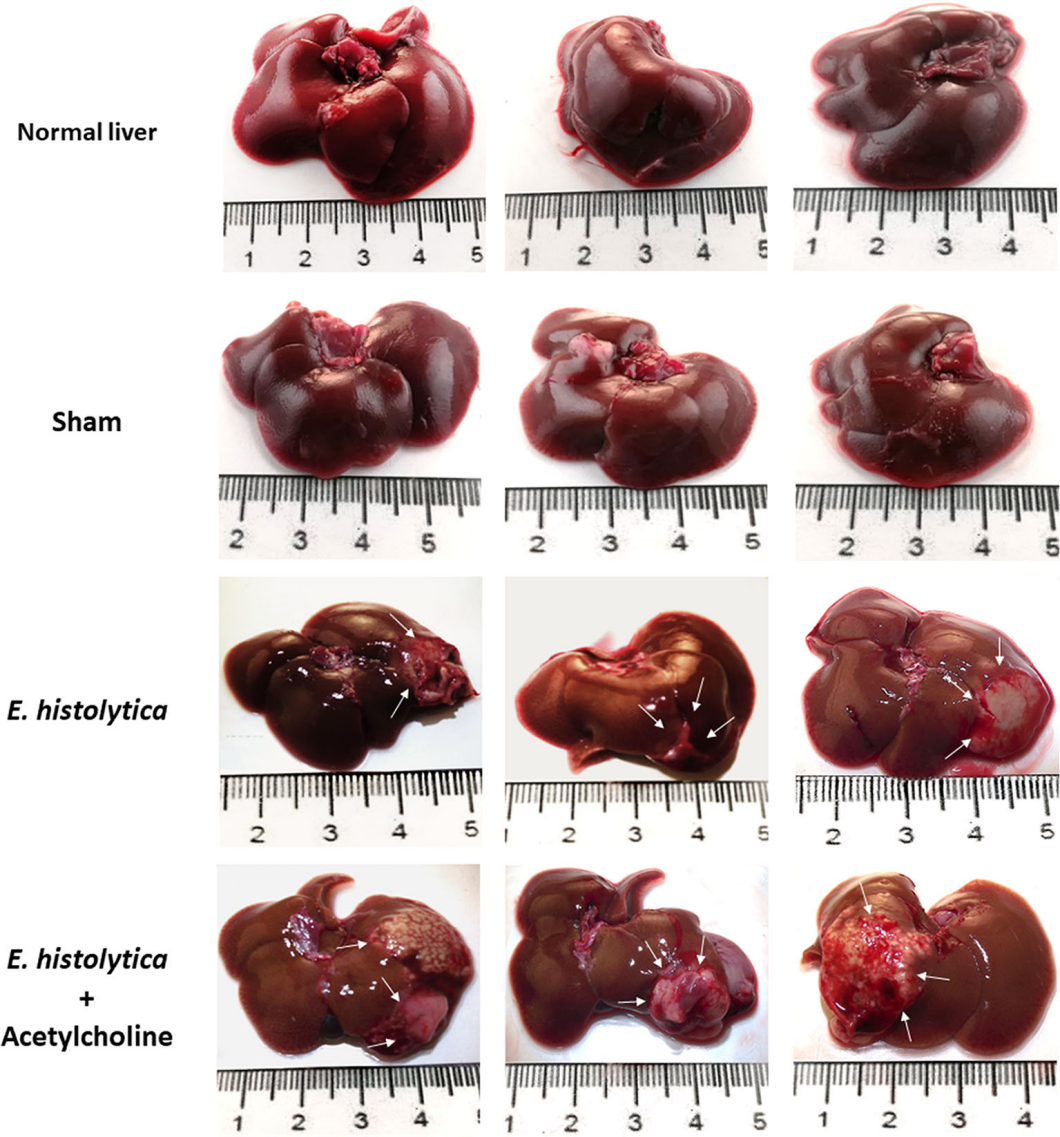
Acetylcholine effects on the development of amebic liver abscesses (ALA). Trophozoites (7.5 × 10^5^) were treated with 0.0001 µM ACh for 1 h and inoculated in hamsters. Representative images of ALA after 4 days of inoculation were taken to show macroscopic damage in male hamsters. Normal liver, control from non-infected hamsters. Sham (inoculated with SFM) and damage produced by ACh-untreated *E. histolytica* trophozoites. Arrows show single lesions by *E. histolytica*, as well as granulomas caused by trophozoites pre-treated with 0.0001 µM ACh.

**Figure 11:**
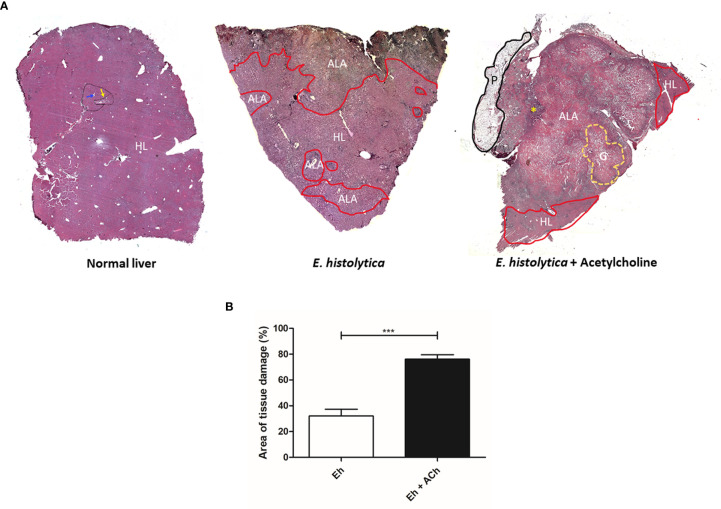
Damage of hepatic tissue caused by acetylcholine-activated E. histolytica trophozoites. **(A)** Histological structure of a hamster liver with normal hepatic characteristics (black line), delimited by portal triads (yellow arrow) and the presence of centrilobular veins (blue arrow). Liver damaged by E. histolytica. The area of healthy liver tissue is located at the central part of the histological section. Areas damaged by trophozoites (red line) located in the periphery, representing 32.01 % ± 18.4 of the liver. Liver damaged by ACh-treated trophozoites. The area of damage is very wide, covering about 76.1 % ± 7.5 of the analyzed tissue. In addition, granulomas (yellow line) and large areas of necrosis with hemorrhage are shown on the left side, an area of abscess opening (yellow asterisk) was observed, and the adjacent peritoneum (black line) formed a barrier likely avoiding the dissemination of purulent content. HL, Healthy Liver; ALA, Amebic Liver Abscess; G, Granuloma; P, Peritoneum. **(B)** Percentage of tissue damage on hamster livers by E. histolytica and E. histolytica treated with ACh. The statistical analysis was performed with Student's t test, where the values of ***p < 0.001 were considered significant.

To evaluate the *in vivo* effect of *E. histolytica* stimulation with ACh, the development of amebic liver abscess (ALA) in hamsters was examined. The control group inoculated with untreated trophozoites presented the characteristic lesions produced by *E. histolytica* after 4 d, that is, small and white lesions localized to the inoculation site in the left liver lobe. In contrast, ACh-treated trophozoites produced several granulomas, a typical lesion caused by *E. histolytica* in hamster liver (Tsutsumi et al., 1984) (**Figure 10**), and histopathologic analysis (**Figure 11**) highlighted a larger lesion and inflammatory infiltration zones with oversized necrotic areas. It was also noticeable the presence of an abscess rupture site in which the spread of purulent material seems prevented by the peritoneum (**Figure 11A**). Furthermore, morphometric analysis showed significantly larger areas of tissue damage produced by *E. histolytica* after ACh stimulation, as compared to those lesions produced by non-treated trophozoites (**Figure 11B**). Altogether, macroscopic, and microscopic tissue analysis demonstrated that ACh promotes the ability of trophozoites to spread, resulting in larger abscesses.

## Discussion

The enteric nervous system, through the release of ACh in response to intestinal inflammation, can regulate diverse gastrointestinal functions, including epithelial ion transport, macromolecular permeability, and intestinal immunity (Gershon and Erde, 1981; Wood, 2008). Evidence suggests that intestinal invasion by trophozoites is primarily promoted by the loss of intestinal barrier integrity and intestinal inflammation during infection. Consequently, ACh could be implicated in the establishment of invasive amebiasis (Leon-Coria et al., 2020). In the present study, our results demonstrated that ACh binds to the trophozoites membrane and, as a consequence, it increases parasite proliferations and chemotaxis, inducing cytoskeleton remodeling, upregulating the expression and secretion of virulence factors, such as Gal/GalNAc lectin, L220, amebapore C and CP, thus, enhancing the ameba capability of host tissue destruction and invasiveness.

Previous *in vivo* studies showed that ACh could be related to *E. histolytica* pathogenicity, without disclosing the mechanisms involved in the regulation of parasite virulence. Indeed, increase ACh levels in rats treated with physostigmine and inoculated with *E. histolytica* trophozoites induced a pathogenic behavior in three out of five *E. histolytica* strains, resulting in a higher cecal lesion scoring (Diamond et al., 1978). Aside of this, there are no other reports that have analyzed the effects of ACh *in vitro* and *in vivo*. Therefore, in the present work, we have studied the hypothesis that ACh can modulate amebic pathogenicity.

It is widely known, that *E. histolytica* can respond to diverse humoral factors like IL-8, IFN-γ, TNF-α, and IL-1β (Zhang et al., 2000; Pfaff et al., 2003; Blazquez et al., 2008; Pulido-Ortega et al., 2019), modulating its capability to cause damage. In this sense, for the first time we are reporting a direct response of the ameba to a neurotransmitter, throughout the binding of ACh to *E. histolytica* trophozoites membrane. This interaction does not affect the parasites viability but stimulates parasite proliferation at physiological ACh intestinal concentrations (Watanabe et al., 1986; Beckmann and Lips, 2013); a similar effect has been described in other organisms (Amaroli, 2017). We observed that ACh treatment activated *E. histolytica* trophozoites and significantly upregulated the expression of virulence factors, including the *Gal/GalNAc lectin* heavy subunit, *amebapore C*, and *ehcp-a2* and *ehcp-a5* cysteine proteases. The Gal/GalNAc lectin plays a key role in *E. histolytica* adherence, cytolysis, and phagocytosis (Saffer and Petri, 1991; Cheng et al., 1998). Likewise, CP of *E. histolytica* are considered important pathogenicity mediators (Bruchhaus et al., 2003). Certainly, these proteolytic enzymes are essential in ALA formation (Stanley et al., 1995; Matthiesen et al., 2013). In particular, EhCP-A5 is directly involved in tissue damage and invasion through the degradation of extracellular matrix components; additionally, EhCP-A5 participates in the evasion of the host immune response by antibodies degradation and inhibition of the complement cascade (Freitas et al., 2009; Hou et al., 2010). On the other hand, EhCP-A2 and amebapores are implicated in cytopathic activity; the latter are pore-forming channels exerting cytopathic and cytolytic activities that are indispensable in host tissue damage and abscess formation (Matthiesen et al., 2013). Altogether, the data presented here suggested that ACh stimulation induces the expression of molecules essential for the parasite’s pathogenic behavior, thereby promoting amebic adherence, phagocytosis, chemotaxis, cytopathic and cytotoxic activities. However, the mechanisms involved in such regulation of gene expression are still under investigation. Their elucidation would help to understand how genome plasticity enables the effective adaptation of ameba to environmental changes.

In addition to this, CP activity was increased by ACh and this could be directly associated to the upregulated expression of *ehcp-a2* and *ehcp-a5*. Furthermore, ACh induced an increased expression of both membrane proteins L220 and Gal/GalNAc, and both participate in trophozoite adherence (Aguirre García et al., 2015), a mechanism closely related to cytoskeleton rearrangement (Manich et al., 2018). It is well known that ACh participates in the activity of the contractile vacuole of the ameba *A. proteus*, a process dependent on cytoskeleton remodeling (Bagrov and Manusova, 2011). In relation to this, our results showed that trophozoites increase the expression of β-actin and augment the polymerization of actin (F-actin) in response to physiological ACh concentrations. The interaction of *E. histolytica* trophozoites with ACh induced cytoskeleton reorganization, as shown by the finding of F-actin-rich structures such as F-actin dots, macropinosomes, and adhesion plates, which were significantly more abundant *in vitro*. During infection, the dynamics of *E. histolytica* cytoskeleton are essential for cellular processes such as adhesion, migration, phagocytosis, and host cell killing (Tavares et al., 2005; Manich et al., 2018). *E. histolytica* killing of host cells include contact-dependent and contact-independent mechanisms. ACh upregulation of virulence factors, in particular soluble secreted components, significantly increased cytotoxicity of ACh-treated trophozoites against HepG2 cell monolayers through contact-independent processes, as evidenced by the increased expression of amebapore and CP, and their activities. ACh stimuli also augmented HepG2 monolayer cytopathic damage (contact-dependent), a process tightly associated to trogocytosis and phagocytosis. Certainly, erytrophagocytosis is a hallmark of amebic pathogenicity (Talamás-Lara et al., 2014) that includes adhesion, cytoskeleton rearrangements, soluble factors secretion mediating host cell killing, and ends with cell debris ingestion. The augmented erythrophagocytosis activity in ACh treated trophozoites is supported by the increment of Gal/GalNAc and L220 protein expression. Consistently, amebic motility, chemotaxis and migration were also augmented after ACh treatment. Our observations suggest that increasing host ACh concentrations could promote the *E. histolytica* invasive behavior during parasite infection. Although the effect of ACh on *E. histolytica* pathogenicity has not been previously studied, former reports provided indirect evidence of the involvement of ACh-activated signaling pathways involving small GTPases, which are considered master regulators of the actin cytoskeleton, and whose activation is linked to cell surface receptors (Tavares et al., 2005; Manich et al., 2018). Specifically, EhRho1, a member of the Rho GTPase family involved in actin polymerization, is particularly active during invasive behavior, thereby promoting amebic motility, increasing migration, and modulating erythrophagocytosis (Franco-Barraza et al., 2006; Spiering and Hodgson, 2011; Bosch and Siderovski, 2013). Similarly, we found that ACh treatment enhanced cytoskeleton reorganization, amebic phagocytic capability, and parasite migration. Altogether, these findings suggest that trophozoites respond to ACh. However, further studies are needed to describe the implied signaling mechanisms, activators, and effectors.

Consistently, ACh also enhanced *E. histolytica* virulence during ALA formation, since exposure of trophozoites to ACh resulted in magnified tissue damage and abscess lesion spreading, producing greater lesion size with an important presence of inflammatory cell infiltrates, and multiple granulomas. Previous reports demonstrated that ACh enhances *E. histolytica* pathogenicity, without disclosing the mechanisms involved in the regulation of parasite virulence. Additionally, during *E. histolytica* infection, patients present increased levels of ACh concentrations in blood which are re-established to normal ranges after treatment (Banu et al., 2005). In accordance with the present work, these previous reports supported the hypothesis that ACh can modulate amebic pathogenicity, favoring the establishment of an invasive disease.

Therefore, the presence of this neurotransmitter at the infection site may affect the efficiency of the host in eliminating the parasite. ACh is constantly synthesized and released by intestinal epithelial cells (Klapproth et al., 1997) and vagus nerve endings, thus controlling visceral functions and the intestinal immune response. During tissue injury, the inflammatory reflex activates a cholinergic anti-inflammatory pathway (Borovikova et al., 2000; Pavlov and Tracey, 2012), increasing the local ACh concentration. This results in a diminished production of pro-inflammatory cytokines by the inhibition of the NF-κB transcription factor and the activation of the anti-inflammatory cytokines through JAK2-STAT3 signaling pathway (de Jonge et al., 2005; Altavilla et al., 2006). Therefore, the presence of ACh at the infection site could hinder *E. histolytica* clearance by suppressing the immune response against the ameba and result in excessive host tissue damage, favoring the dissemination of the infection.

In conclusion, this is the first report of the ability of *E. histolytica* to bind ACh, and ACh regulation of amebic virulence factors. Our findings showed that *E. histolytica* trophozoites can bind ACh on their membrane, thereby modulating amebic virulence by inducing cytoskeleton rearrangement, parasite motility, and phagocytosis, as well as by increasing the expression and secretion of virulence factors, and consequently promoting amebic cytotoxicity. Based on these findings, further studies are needed to understand the molecular mechanisms involved in the modulation of *E. histolytica* virulence in response to ACh during parasitic colonization, which can potentially influence the progression and severity of amebiasis.

## Data Availability Statement

The raw data supporting the conclusions of this article will be made available by the authors, without undue reservation.

## Ethics Statement

The animal study was reviewed and approved by Committee on Bioethics in the animal facilities of the Autonomous University of Aguascalientes.

## Author Contributions

VJ, MO and MRMN conceived and design the experiments. MRMN performed experiments, data analysis and wrote manuscript. MH and SM performed the experimental amebic liver abscess in hamsters, and histological sample processing and analysis. MH and MRB western blot assays. TR production and purification of anti-L220. AB performed image and statistical analysis. VJ, MO, GH and MRIE contributed to writing and revising the manuscript. All authors contributed to the article and approved the submitted version.

## Funding

This work was supported by the PIBB16-2 grant of the Universidad Autónoma de Aguascalientes for JV-J, the CONACYT grant for JV-J (286184), and the CONACYT Doctoral fellowship for MM-R (Number CVU: 555426).

## Conflict of Interest

The authors declare that the research was conducted in the absence of any commercial or financial relationships that could be construed as a potential conflict of interest.

## Supplementary Material

Supplementary Video 1 shows motility of E. histolytica and their erratic movement in short distances, there is increase movement towards ACh 0.0001 µM, located in bottom central point of video, compared to control CD, SFM, serum, and ACh0.01 µM, 0.000001 µM (**Figure 9C**).
